# Herbal Medicines for the Treatment of Chronic Obstructive Airway Diseases (Asthma or Chronic Obstructive Pulmonary Disease): A Prospective Observational Study

**DOI:** 10.1155/2022/3485757

**Published:** 2022-05-29

**Authors:** Yee Ran Lyu, Su-Won Lee, Si-Yeon Kim, Hye-Bin Han, Won-Kyung Yang, Seung-Hyung Kim, In Chul Jung, O-jin Kwon, Ae-Ran Kim, Jinhee Kim, Mi Young Lee, Yang-Chun Park

**Affiliations:** ^1^Korean Medicine Science Research Division, Korea Institute of Oriental Medicine, Daejeon, Republic of Korea; ^2^Division of Respiratory Medicine, Department of Internal Medicine, College of Korean Medicine, Daejeon University, Daejeon, Republic of Korea; ^3^Clinical Trial Center, Daejeon Korean Medicine Hospital, Daejeon University, Daejeon, Republic of Korea; ^4^Immunotherapy Research Center, Korea Research Institute of Bioscience and Biotechnology, Daejeon, Republic of Korea; ^5^Department of Neuropsychiatry, College of Korean Medicine, Daejeon University, Daejeon, Republic of Korea; ^6^Clinical Medicine Division, R&D Strategy Division, Korea Institute of Oriental Medicine, Daejeon, Republic of Korea; ^7^Korean Medicine Convergence Research Division, Korea Institute of Oriental Medicine, Daejeon, Republic of Korea

## Abstract

**Background:**

Obstructive airway disease is a major health problem and has a great impact on global socioeconomic burden. Despite therapeutic advances in recent decades, there is still a need for effective and safe therapeutic agents for patients with asthma or chronic obstructive pulmonary disease (COPD).

**Methods:**

This prospective observational study explored the effects of herbal medicines in patients with asthma and COPD. All participants visited the hospital at least every 4 weeks for 12 weeks to receive their herbal medicines based on their pattern identification and to evaluate safety and efficacy endpoints. We followed the diagnostic criteria used by Korean medicine doctors to prescribe herbal medicines, explored variations in prescribed herbal medicines, and explored a number of clinical features in patients with asthma or COPD.

**Results:**

A total of 24 patients were enrolled: 14 were diagnosed with asthma and 10 with COPD and 19 completed the study. After 12 weeks of herbal medicine treatment, herbal medicines significantly improved the modified Clinical Asthma Measurement Scale in Oriental Medicine-V in asthma patients and the modified Medical Research Council Dyspnoea Scale and St. George's Respiratory Questionnaire in COPD patients. For all patients, modified Medical Research Council Dyspnoea Scale score and interleukin-13 were found to be significantly different after treatment. Additionally, the majority of patients were satisfied with our herbal medicine treatments, and no severe adverse events were reported during the study.

**Conclusions:**

Our study provides preliminary clinical data on the safety and efficacy of herbal medicines in patients with asthma and COPD.

## 1. Introduction

Asthma and chronic obstructive pulmonary disease (COPD) are the most prevalent obstructive airway diseases associated with chronic inflammation and airway obstruction [1]. Despite therapeutic advances in recent decades, both diseases are increasing in prevalence and morbidity, affecting more than 500 million people worldwide [2]. The global prevalence of asthma in adults is estimated to be 4.3% [3], and the prevalence of COPD is 11.7% [4]. In addition, these chronic respiratory diseases have significant adverse effects on quality of life, disability, and productivity, thereby increasing the social and economic burden [5]. Thus, effective therapeutic strategies for asthma and COPD are essential to improve the physical and psychological well-being of patients and reduce the socioeconomic burden of respiratory disease.

Asthma is defined by a history of respiratory symptoms such as wheezing, shortness of breath, chest tightness, cough, and variable airflow limitation. For the current treatment for asthma, bronchodilators and anti-inflammatory medications are primarily used to control symptoms and to reduce the risk of asthma [6]. However, only half of patients with asthma respond adequately to current therapies [7]. COPD also presents with symptoms similar to those of asthma, such as chronic cough, excess sputum production, chest tightness, and shortness of breath during physical activity, which is usually caused by significant exposure to noxious particles or gases. Pharmacological therapies, including long-acting bronchodilators and inhaled corticosteroids (ICS), are used to reduce the symptoms, frequency, and severity of exacerbations, and to improve physical activities, although there is no conclusive evidence of long-term decline in lung function [8]. Therefore, these unsatisfactory treatment outcomes and adverse effects from conventional medications contribute to the medical needs for additional effective and safe therapeutic agents for asthma or COPD.

Over the past decades, complementary or alternative medicine (CAM), including herbal medicines, has been widely used for the treatment of asthma and COPD, with little risk and few complications. A previous study reported that 52% of patients with asthma and 33% with COPD had used CAM before [9]. Another US survey also found that allergies and lung problems were some of the most frequent medical conditions where CAM had been used before [10]. Recently, several systematic reviews have been conducted to determine the effectiveness of these herbal medicines and found the potential effectiveness of herbal medicines in both asthma and COPD with beneficial anti-inflammatory, antioxidant, immunostimulatory, antimicrobial, cough suppressant, smooth muscle relaxant, and expectorant effects [11]. However, the efficacy of CAM remains inconclusive due to the low quality of studies, and further studies are needed to identify novel therapeutic herbal medicines for asthma and COPD.

Therefore, in this study, we investigated the effects of various herbal medicines on asthma and COPD in real-world clinical practice at the Korean Medicine Hospital to identify candidates for novel therapeutic agents. We observed the diagnostic criteria used by Korean medicine doctors to prescribe herbal medicines, variations in prescribed herbal medicines, and diverse clinical features, including respiratory symptoms, lung function, quality of life, and physical activities in patients with asthma or COPD. We expect that our findings will be used as a basis to find an effective herbal medicine and to develop a well-designed clinical trial to confirm the safety and efficacy of herbal medicines in obstructive airway diseases.

## 2. Materials and Methods

### 2.1. Study Design

This prospective observational study explored the effects of herbal medicines in patients with asthma and COPD. The study was conducted at the Daejeon Korean Medicine Hospital of Daejeon University between 26 February 2019 and 4 December 2020. A total of 24 patients diagnosed with asthma or COPD were enrolled, and 19 completed the study. After participants were determined to be eligible based on the inclusion and exclusion criteria and voluntarily signed a written consent form, they were prescribed appropriate herbal medicines based on pattern identification diagnosis by a professional clinician of Korean medicine. All participants were asked to visit the hospital at least every 4 weeks (week 0, week 4, week 8, and week 12) for 12 weeks to receive their herbal medicines, as well as to evaluate safety and efficacy endpoints. During the study period, participants were allowed to visit the hospital as often as they needed and receive other Korean medicine treatment options, such as acupuncture, pharmacoacupuncture, moxibustion, cupping, and physical therapies or Western medications. All of these concomitant treatments were recorded, and all data were coded confidentially.

The study was conducted in accordance with the Declaration of Helsinki and the Good Clinical Practice guidelines. This study was approved by the Institutional Review Board of the Daejeon Korean Medicine Hospital of Daejeon University (DJDSKH-18-BM-14) and registered at the Clinical Research Information Service (KCT0004919).

### 2.2. Participants

Participants aged 19 years who were diagnosed with asthma according to the Global Initiative for Asthma (GINA) standard [6] or COPD in the Global Initiative for Chronic Obstructive Lung Disease (GOLD) standard [8] were enrolled in our study. Those who met any of the following criteria were excluded: presence of severe respiratory disease other than asthma or COPD (for example, cystic fibrosis, pneumonia, interstitial lung disease, or lung cancer); history of abuse or dependence on alcohol or other substances; history or presence of clinically relevant cardiovascular, renal, metabolic, haematological, neurological, psychiatric, systemic, or infectious disease or malignant tumor (except in patients with no evidence of tumor recurrence for more than 5 years after surgery); disease that can affect drug absorption or digestive disorders after related surgery; history of hypersensitivity and allergies to research-related drugs; pregnancy or breastfeeding; or patients judged by the investigators to be inappropriate for inclusion in the clinical study.

### 2.3. Herbal Medicine Treatment

Herbal medicines for each participant were prescribed according to the pattern identification, a diagnostic criterion to determine the treatment methods based on the patients' clinical data by a professional clinician in Korean medicine. At baseline, the professional clinician determined the pattern identification of each participant by their disease based on the clinical symptoms and signs, among the pattern types previously developed for asthma and COPD. The pattern identification for asthma consists of lung deficiency, wind-cold, heart-kidney deficiency, upper excess and lower deficiency, phlegm-dampness stagnation, phlegm-heat, and cold-phlegm [12], and the pattern identification of COPD is divided into wind-cold, phlegm turbidity, lung-heat, lung deficiency, spleen deficiency, kidney-yang deficiency, and kidney-yin deficiency [13]. The major symptoms and signs used for the assessment of pattern identification include the symptoms of cough, dyspnoea, sputum, rhinorrhoea, chest pain, and throat discomfort.

According to the determined pattern identification, participants were prescribed personalized herbal medicines for the next 4 weeks. For each pattern identification type in asthma and COPD, specific herbal medicines that responded to each pattern were prescribed. All participants were then reevaluated for pattern identification at every visit, and the herbal medicines being prescribed were maintained or changed by their newly determined pattern types for the next 4 weeks. The number of doses of each herbal medicine was also determined by the disease status of each participant, two to three times daily. All herbal medicines prescribed in this study were manufactured by a pharmaceutical company that obtained authorization from the Korea Good Manufacturing Practice.

### 2.4. Efficacy Outcome Measures

For the efficacy outcome measures in this study, lung function, respiratory symptom scales, health-related quality of life questionnaire, biomarkers, and patient satisfaction scale were evaluated in all patients with obstructive airway diseases throughout the study.

The pulmonary function test (PFT) and peak expiratory flow rate (PEFR) were assessed to evaluate the changes in airway obstruction after herbal medicine treatments compared to the baseline. The PFT, including forced volume capacity (FVC), forced expiratory volume in 1 s (FEV1), and FEV1/FVC, was measured at baseline and week 12 using a spirometer (Vmax 20 system; SensorMedics, Yorba Linda, CA, USA), according to the PFT guidelines established by the Korean Academy of Tuberculosis and Respiratory Diseases [14]. Additionally, more readily available PEFR (MicroPeak, CareFusion, Basingstoke, UK) was assessed every 4 weeks to monitor the degree of reversibility of airflow obstruction [15].

For the assessment of respiratory symptoms, the modified Clinical Asthma Measurement Scale in Oriental Medicine-V (mCAMSOM-V), COPD assessment test (CAT, for COPD patients only), asthma control test (ACT, for asthma patients only), modified Borg Scale, and modified Medical Research Council (mMRC) Dyspnoea Scale were used. The mCAMSOM-V is a modified version of the CAMSOM-V which was suggested as the primary outcome measure in the Traditional Korean Medicine Clinical Practice Guidelines for antiasthmatic agents [12]. We added some questions related to the color and amount of sputum to make it useful in both asthma and COPD, consequently consisting of the symptom scales of cough, wheezing, sputum, dyspnoea, daily activities, and night-time sleep disturbance. More specific assessment tools for asthma and COPD were evaluated by CAT for COPD patients and ACT for asthma patients, both of which are recommended by GOLD or GINA for the assessment of each respective disease. CAT is a self-administered, eight-item health status instrument to assess the symptoms and impacts of COPD [16], and ACT is a patient-report, validated, five-item questionnaire for identifying patients with poorly controlled asthma [17]. In addition, for the measurements of dyspnoea, which is one of the major discomforts that obstructive airway disease patients suffer, the modified Borg Scale and mMRC were measured every 4 weeks. The modified Borg Scale asks patients about the severity of breathlessness, with no breathlessness at all scoring 0 and maximal breathlessness scoring 10 [18], and the mMRC comprises five statements that describe the range of respiratory disability, with lower scores indicating better physical ability due to dyspnoea [19].

Health-related quality of life was measured by the St. George's Respiratory Questionnaire (SGRQ) before and after treatment with herbal medicines. The SGRQ is a validated and widely used dyspnoea-specific quality of life questionnaire for chronic respiratory diseases such as asthma and COPD [20], consisting of symptom, activity, and impact domains, with a total score from 0 to 100, and the validity and reliability of the Korean version of the SGRQ have also been proven [21].

Biomarkers including C-reactive protein (CRP), fibrinogen, neutrophil, tumor necrosis factor-*α* (TNF-*α*), interleukin 4 (IL-4), interleukin 5 (IL-5), interleukin 6 (IL-6), interleukin 13 (IL-13), immunoglobulin *E* (IgE), and eosinophils, which are specific to the inflammatory process of asthma or COPD, were measured at baseline and endpoint to determine if there were any changes in these markers after the administration of herbal medicines. For other additional outcomes, the Integrative Medicine Patient Satisfaction Scale (IMPSS) was measured for all participants at week 12 to assess patient satisfaction after herbal medicine treatments [22].

### 2.5. Safety Outcomes

For safety outcomes, vital signs (blood pressure, pulse, and body temperature), adverse events, and laboratory examinations (liver function test, routine blood tests, and urine tests) were assessed throughout the study. Vital signs and adverse events were observed at every visit, and laboratory examinations were performed before and after treatment.

### 2.6. Statistical Methods

All statistical analyses were performed by an independent statistician using SAS^®^ (version 9.4, SAS Institute, Cary, NC, USA). Continuous variables are presented as mean (95% confidence interval), and categorical variables are reported as frequencies (percentages). Statistical significance was accepted with a two-sided test and an *α* level of 0.05. The missing values were handled using a multiple imputation method. For efficacy outcome measures, Student's paired *t*-test was used.

## 3. Results

### 3.1. Study Participants and Baseline Characteristics

A total of 29 patients with obstructive airway disease were screened, and 24 eligible patients were enrolled in this study. During the study period, 5 patients dropped out due to the withdrawal of informed consent and loss of follow-up. All 24 enrolled participants were included in the full analysis and safety datasets.

The baseline characteristics of the 24 patients are shown in Table 1. Overall, 12 men and 12 women, with an average age of 57.46 years, were included in this study. Among the 24 participants, 14 were diagnosed with asthma and 10 had COPD. The severity of diseases, diagnosed either by GINA or GOLD guidelines, was found to vary from intermittent mild to persistently severe in asthma patients, and from A to C in COPD patients at baseline. The number of patients using bronchodilators or inhaled corticosteroids was found to be 11 in asthma and 5 in COPD patients, as shown in Table 1.

### 3.2. Herbal Medicine Treatment

**Figure 1:**
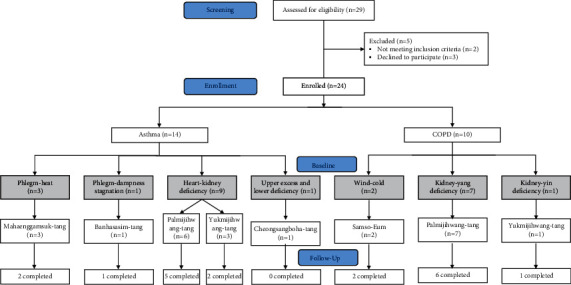
Flow chart for the study subjects.

The herbal medicine treatments were prescribed by the pattern identification of each participant at every visit, and the initial herbal medicines were prescribed by the pattern types diagnosed at baseline, as shown in Figure 1. Among the 14 asthma patients, 9 were included in the heart-kidney deficiency pattern, 3 in the phlegm-heat pattern, 1 in the phlegm-dampness stagnation pattern, and 1 in the upper excess and lower deficiency pattern. In COPD patients, 7 had a kidney-yang deficiency pattern, 2 a wind-cold pattern, and 1 a kidney-yin deficiency pattern. Each participant was then prescribed herbal medicines specific to each of their determined pattern-type groups.

The distribution and flow chart of the prescribed herbal medicines throughout the trial for all participants are shown in Figure 1. There were no changes in pattern identification or herbal medicines during the study period. We found that palmijihwang-tang was the most prescribed herbal medicine for both asthma and COPD patients, followed by yukmijihwang-tang and mahaenggamsuk-tang in patients with asthma, and samsoeum as well as yukmijihwang-tang in COPD patients.

During the trial period, participants were also treated with Western medications (Table 1) and other Korean medicine treatment options, such as acupuncture, pharmacoacupuncture, and moxibustion as shown in Table 2.

### 3.3. Efficacy Outcome Measures

#### 3.3.1. Lung Function

At baseline, the study participants with asthma and COPD obtained results of 67.29% and 54.90%, respectively, in FEV1/FVC, and 65.50% and 79.93%, respectively, in predicted FEV1 value, indicating that the average included participant with asthma and COPD had a moderate degree of disease. After the herbal medicine treatments for 12 weeks, FEV1, FEV1/FVC, and PEFR did not show significant changes in the asthma and COPD groups (Table 3).

#### 3.3.2. Respiratory Symptoms

The mCAMSOM-*V* score significantly decreased from 22.86 at baseline to 20.06 at week 12 in the asthma group (*p*=0.0401), indicating that the overall symptoms of cough, wheezing, sputum, dyspnoea, daily activities, and night-time sleep disturbance had improved after herbal medicine treatments with significant differences. However, the score in the COPD group did not show significant differences, although it showed a decrease from 13.70 to 13.52 after 12 weeks of herbal medicine treatment (Table 4).

Regarding the ACT scale, we found that the score increased from 14.57 at baseline to 16.26 in patients with asthma after 12 weeks of treatment. The average ACT score at baseline indicates that the symptoms of asthma in our study participants were not well controlled, by scoring under the cut-off of 20 points discriminating patients with controlled asthma. Analysis at the individual subject level showed that two of the 14 asthma participants were at a controlled level and the other 12 were at an uncontrolled level at baseline. However, after 12 weeks of treatment, the number of asthma-controlled participants increased to 5 among 14 asthma patients. In the COPD group, the CAT score did not show a significant decrease after herbal medicine treatment, which was calculated by adding the symptom scores of cough, sputum, chest discomfort, dyspnoea, physical activity, and sleep disturbance (Table 5).

**Figure 2:**
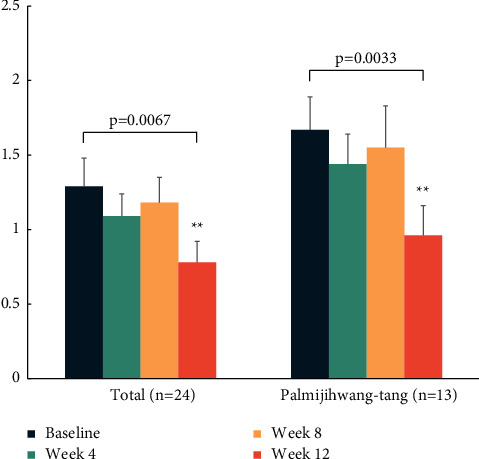
mMRC before and after herbal medicine treatments. Data were presented as mean ± SD (^*∗∗*^*p* < 0.01).

However, we found significant improvement in symptoms of dyspnoea, measured by mMRC, in COPD patients and in all participants after 12 weeks of treatment (*p*=0.0269, 0.0067). The mMRC score in COPD patients gradually decreased from 1.60 at baseline to 1.50 at week 4 and 8, and to 0.89 at week 12 (Table 6). Additionally, it was found that 5 of the 10 COPD patients achieved a cut-off value of ≥2 points in mMRC at baseline, and all of those 5 patients scored less than 2 points after herbal medicine treatments. The mMRC scores of each participant were distributed as follows: 2 patients scored 0, 3 patients scored 1, 2 patients scored 3, and 3 patients scored 4 at baseline; this changed to 2 patients scoring 0 and 7 scoring 1. Additionally, we found a significant improvement in mMRC in participants who had received palmijihwang-tang after 12 weeks compared to the baseline (*p*=0.0033), indicating that palmijihwang-tang is effective in relieving the symptoms of dyspnoea in airway obstructive diseases (Figure 2). The other dyspnoea score of the modified Borg Scale decreased after 12 weeks of administration, from 2.17 at baseline to 2.11 at week 12, although the difference was not significant (Table 6).

#### 3.3.3. Quality of Life

The patients' quality of life, evaluated by the SGRQ, showed significant improvements in COPD patients. The total SGRQ score significantly decreased from 42.87 at baseline to 35.25 at week 12 (*p*=0.0265), and the impact subscale score also significantly decreased from 24.67 to 16.51 after the administration of herbal medicine (*p*=0.0147). The other scores of symptom and activity subscales also showed improvements from 51.95 to 51.99 at baseline to 46.30 and 42.95 at week 12, respectively; however, neither of the changes was statistically significant. The difference in total SGRQ scores was also found to be more than the minimal clinically important difference of 4 in COPD and all participants, being 7.62 points in the COPD patients and 4.12 points in all participants (Table 7).

#### 3.3.4. Biomarkers

The previously reported biomarkers related to inflammation of obstructive airway diseases, CRP, fibrinogen, neutrophil, eosinophil, TNF-*α*, IL-4, IL-5, IL-6, IL-13, and IgE, were evaluated before and after herbal medicine treatment. Among these biomarkers, we found that IL-13 was significantly decreased after 12 weeks in patients with obstructive airway disease. The level of IL-13 at baseline decreased from 27.77 to 15.13 at week 12. No significant differences were found in the levels of other inflammatory cytokines and cells. However, we found that TNF-*α* and IL-6 in both asthma and COPD patients showed a tendency to decrease after administration of herbal medicines, and IgE in the asthma group showed a comparable decrease after 12 weeks (Table 8).

#### 3.3.5. Patient Satisfaction

**Figure 3:**
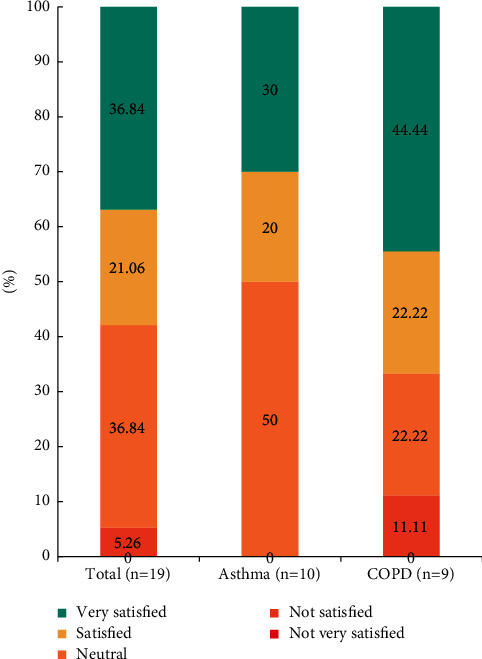
Integrative Medicine Patient Satisfaction Scale (IMPSS) after herbal medicine treatments.

Patient satisfaction after herbal medicine treatment was assessed at week 12 in all participants. Among the 19 patients who had completed the study until the last visit, 36.84% of participants answered that they were “very satisfied” with the treatments, 21.06% answered “satisfied,” 36.84% answered “neutral,” and 5.26% answered “not satisfied.” The patients were generally satisfied with the treatment of herbal medicines for the management of obstructive airway disease (Figure 3).

### 3.4. Safety Outcomes

Safety assessments were conducted with all 24 participants throughout the study period of 12 weeks. Seven adverse events were observed during the study: four in the asthma group and three in the COPD group. None of them had severe adverse events, and no participant dropped out due to the occurrence of adverse events. The reported adverse events included general weakness (2 cases), fatigue (1 case), anorexia (1 case), chest discomfort (1 case), palpitations (1 case), and cellulitis (1 case). Among the seven adverse events, six were determined as mild and one as moderate, with the latter being a case of cellulitis that required prolonged hospitalization. The majority were also considered to be unrelated to the intervention, and two cases of general weakness and palpitations were recorded as probably related to herbal medicine use and were instructed to discontinue the administration of herbal medicine. In the vital signs and laboratory tests, there were no significant differences or clinically significant values during the study period.

## 4. Discussion

Obstructive airway disease is a major health problem and has a great impact on global socioeconomic burden. Diseases classified as obstructive airway diseases, asthma and COPD, have overlapping clinical features and frequently coexist. Both diseases exhibit similar respiratory symptoms, such as cough, wheezing, sputum, and chest discomfort, and both are mostly managed by bronchodilators and inhaled corticosteroids [23]. The herbal medicines used for asthma and COPD are also similar, aimed at inhibiting airway inflammation and relieving respiratory symptoms. Moreover, it was reported that asthma patients who used herbal medications were less likely to visit emergency department [24] and were more likely to be experiencing lower quality of life than people who do not use these treatments [25], indicating the potential effect of herbal medicines on asthma-related ED visits. Therefore, we investigated the effects of various herbal medicines used for both obstructive airway diseases, asthma and COPD, in our study on Korean medicine clinical practice, as their prescriptions often overlap.

In previous studies, several herbal medicines have been reported for their efficacy in asthma and COPD, such as maekmoondong-tang (Mai-Men-Dong-Tang), yukmijihwang-tang (Liu-Wei-Di-Huang-Wan), samryungbaekchul-san (Shen-Ling-Bai-Zhu-San), gami-sagunja-tang (Jai-Wei-Si-Jun-Zi-Tang), jeongcheon-tang (Ding Chuan Tang), and sibak-tang (saiboku-to) for asthma and geumsuyukgunjeon (Jinshui Liujun decoction), gami-okbyungpoongsan (Jiawei Yupingfeng), bojungikki-tang (hochuekkito), and ginseng (Panax ginseng extract capsule) for COPD. However, the efficacy of herbal medicines still lacks evidence due to the low quality of studies, and we found no prior clinical trials using herbal medicines for asthma or COPD performed in South Korea. Thus, we investigated the use of various herbal medicines for patients with obstructive airway diseases in Korean medicine clinical practice. Notably, in our study, we focused on herbal medicines which are manufactured by the pharmaceutical company as a formula, since our study aimed to find a candidate for a novel therapeutic remedy which can be used worldwide in obstructive airway diseases.

In our study, a total of 24 patients were enrolled, with 14 being diagnosed with asthma and 10 with COPD, and 19 completed the study. For 12 weeks, participants were administered herbal medicines, which were prescribed based on their pattern identification as diagnosed by professional clinicians. Professionals of Korean medicine diagnose the patterns of patients according to their symptoms and signs related to the disease. Moreover, pattern identification tools have been developed for each disease in an attempt to achieve objectivity. Both the pattern identification questionnaires for asthma and COPD were also developed previously, although they have not yet been validated. In our study, patients were diagnosed using their patterns both by professional and pattern identification questionnaires, although herbal medicines were actually prescribed by the professional as questionnaires were not validated yet and are still not widely used in clinical practice. When we compared the diagnosed pattern identifications between professionals and questionnaires after the study, we found inconsistent patterns. In asthma, patients were categorized as follows: 1 as wind-cold, 5 as phlegm-dampness stagnation, 3 as heart-kidney deficiency, 4 as upper excess and lower deficiency, and 1 as lung deficiency on pattern identification questionnaire. This was in contrast to the results from the professionals, where 3 were classified as phlegm-heat, 1 as phlegm-dampness stagnation, 9 as heart-kidney deficiency, and 1 as upper excess and lower deficiency. Similarly, in COPD, patients were classified as follows: 2 as wind-cold, 3 as kidney-yang deficiency, 2 as kidney-yin deficiency, 2 as phlegm-dampness stagnation, and 2 as lung deficiency on questionnaires; this is in contrast to the distribution of 2 as wind-cold, 7 as kidney-yang deficiency, and 1 as kidney-yin deficiency determined by the professional. These inconsistent results indicate that further studies are needed to develop reliable and useful pattern identification tools for asthma or COPD that are highly correlated with clinical practice.

Moreover, we obtained information on the types of frequently used herbal medicines in patients with asthma and COPD. Palmijihwang-tang was the most prescribed herbal medicine for both asthma and COPD patients, accounting for 13 cases among a total of 24 cases. Then, yukmijihwang-tang and mahaenggamsuk-tang were followed by asthma patients and samsoeum and yukmijihwang-tang by COPD patients. We believe that these reported medicines can be considered candidates for our next confirmatory trial to evaluate the safety and efficacy of herbal medicine in obstructive airway diseases.

For the assessment of herbal medicines in the treatment of obstructive airway diseases, various outcomes including PFTs, respiratory symptom scales, QoL, and inflammatory biomarkers were observed throughout the study period. Based on the clinical outcomes at baseline, both asthma and COPD patients enrolled in our study were revealed to have a moderate degree of airway obstruction, poor health status, and quality of life, while the symptoms of dyspnoea were not symptomatic. In the pulmonary function tests, airway obstruction was found to be more severe in COPD patients than in asthma patients, as 65.50% was recorded for the predicted FEV1 value in COPD patients and 79.93% in asthma patients. Additionally, asthma patients showed uncontrolled levels of their disease by scoring 14.57 in the ACT, and COPD patients also showed poor health status by scoring 17.70 CAT, with the cut-off value being 10 in the CAT [26]. For both asthma and COPD patients, poor quality of life was observed by recording 49.94 and 42.87, respectively, in the SGRQ, which exceeded the cut-off value of 25 points [27]. However, the symptom of dyspnoea was found to be not symptomatic, as participants scored less than the cut-off value of 2 in mMRC [28] and 4 to 6 in the modified Borg Scale in our study [29].

After 12 weeks of herbal medicine treatments, we found significant improvements in respiratory symptoms in asthma patients, assessed by mCAMSOM-V, and symptoms of dyspnoea and quality of life in COPD patients, as measured by mMRC and SGRQ. For all participants with asthma and COPD, mMRC and IL-13 were found to have significant differences after treatment. Analysis by each herbal medicine prescribed for participants showed that palmijihwang-tang had significant efficacy in relieving dyspnoea by improving the mMRC score for patients with obstructive airway disease. Additionally, it was found that the majority of patients were satisfied with our treatment with herbal medicines, and no severe adverse events were reported during the study.

In asthma patients, herbal medicines have shown significant efficacy in mCAMSOM-V, which evaluates patients' overall symptoms of cough, wheezing, sputum, dyspnoea, daily activities, and night-time sleep disturbance. These results demonstrate that herbal medicines have beneficial effects in the treatment of asthma by controlling respiratory symptoms, which is the major goal in the management of asthma. Moreover, as mCAMSOM-V was originally developed for the evaluation of asthma and is suggested as the primary outcome measure in the Traditional Korean Medicine Clinical Practice Guidelines for antiasthmatic agents, our finding that herbal medicines have shown significant efficacy in mCAMSOM-V is worthwhile. On the other asthma control outcome measure, ACT, a significant improvement was not found after the herbal medicine treatments. However, we found that the number of patients included in the control level of asthma increased from 2 at baseline to 5 at week 12. Overall, the severity of asthma was also found to be improved in four patients, by stepping up from persistent mild to intermittent mild in 3 and from persistent moderate to persistent mild in 1, while 6 had no change in their severity and 4 had dropped out.

For COPD patients, significant efficacy of herbal medicines was found when assessed using the mMRC and SGRQ. Dyspnoea is the most common and debilitating symptom experienced by patients with COPD, which also reduces the quality of life and physical activities [30]. The level of dyspnoea also provides important prognostic information and is suggested as a better predictor of 5-year survival than airway obstruction in patients with COPD [31]. Thus, our study results showing significant improvements in dyspnoea after treatment with herbal medicines are valuable, as they show the efficacy of herbal medicines with the most important prognostic factor in the management of COPD. We also found significant improvement and clinically meaningful differences in SGRQ after treatment with herbal medicines in COPD patients, which correlate with our positive results for dyspnoea. The mMRC has been previously reported to have strong correlations with the SGRQ and is a good indicator of health-related quality of life in patients with COPD [32]. However, we could not find a significant effect of herbal medicines on other respiratory symptom scales, including mCAMSOM-V and CAT, indicating that herbal medicines are expected to have beneficial effects on COPD by relieving breathlessness rather than other clinical symptoms. Regarding the severity of COPD based on the GOLD guidelines, two patients were found to improve, one from B to A and the other from C to B.

Moreover, we found a significant decrease in the level of IL-13 in both asthma and COPD patients, which was measured as a biomarker of obstructive airway diseases in our study. Over the past few years, compelling evidence of IL-13 as a key mediator and critical effector molecule in the inflammatory processes of asthma and COPD has been established [33]. Inhibition of IL-13 activity has been reported to prevent airway hyperresponsiveness, pulmonary eosinophilia, and mucus production [34]. Our finding of inhibiting the level of IL-13 by herbal medicines may represent a new mechanism by which herbal medicines act on the inflammatory process of obstructive airway diseases.

Overall, we revealed the potential of herbal medicines for obstructive airway diseases as an effective and safe remedy. We could also see the possibility of a therapeutic effect on palmijihwang-tang, which had shown efficacy in relieving dyspnoea and was found to be the most prescribed formula in our study. Despite these beneficial effects of herbal medicines in patients with asthma and COPD, our findings need to be interpreted in light of a few limitations. First, our study was conducted with a small sample size. Although the aim of our study was not to firmly conclude the effectiveness of herbal medicines, it was difficult to find significant differences and estimate the effects of our treatment precisely with a relatively small number of patients. We could not confirm a significant efficacy of typical herbal medicines except for the mMRC results, and we believe that a larger number of patients will be needed to address this. As a small study, there are limitations in concluding reliable and precise results, due to the problems with a large standard error, false-positive errors, and statistical analyses [35], and thus our study results can also provide only preliminary data on the efficacy of herbal medicines in obstructive airway disease. Larger confirmatory clinical trials will be required to determine the safety and efficacy of herbal medicines based on our study results. Second, as our study was an observational study without a control group, the clinical data were analyzed only before and after comparison, and we need to consider potential biases associated with observational studies, such as confounding bias, information bias, and selection bias. Contrary to randomized clinical trials, observational studies cannot completely exclude the possibility of residual confounding factors. In our study, we cannot ignore the effects of other treatment methods which were allowed during the study period or the placebo effects of herbal medicines, as we did not restrict the other treatment options for managing obstructive airway disease and as our study was designed as single group, before-after study. We found that 11 patients out of 14 asthma patients and 5 out of 10 COPD patients were using bronchodilators or ICS during the trial period and most patients were treated with acupuncture, moxibustion, and pharmacoacupuncture when visiting hospital. Nevertheless, observational studies, such as our study, can provide real-world clinical data and address important clinical questions, which is difficult in association with the rigor and strict selection process of randomized controlled trials. We obtained clinical data related to the process of diagnosing pattern identification, types of herbal medicines being frequently prescribed, and various outcomes related to obstructive airway diseases in Korean medicine clinical practice. We expect that our study will be used as preliminary data for the development of confirmatory clinical trials of herbal medicines, in the context of selecting the candidate herbal medicines and designing the protocol of clinical trials including inclusion/exclusion criteria, intervention period, and outcome measures, which might not have received sufficient clinical data to support the design without the existence of our observational study.

## 5. Conclusion

Our study provides preliminary clinical data on the safety and efficacy of herbal medicines in patients with asthma and COPD. We obtained information on the distribution of pattern identification and herbal medicine prescription for obstructive airway disease, which will be used for deciding the herbal medicine candidate for a subsequent confirmatory clinical trial. We also found the potential effects of herbal medicines in relieving overall respiratory symptoms in asthma and improving the symptoms of dyspnoea and quality of life in COPD patients, as well as inhibiting IL-13 levels in both asthma and COPD patients, with a high level of patient satisfaction. We expect that our findings will be used as a basis to establish a well-designed, large-scale, confirmatory clinical trial to evaluate the safety and efficacy of herbal medicines in obstructive airway diseases.

## Figures and Tables

**Table 1:** Baseline characteristics.

Characteristic	Total (*n* = 24)	Asthma (*n* = 14)	COPD (*n* = 10)
Age (year)^†^	57.46 (51.89, 63.03)	52.07 (44.66, 59.48)	65.00 (57.92, 72.08)
Gender (M/F)^‡^	12 (50.00%)/12 (50.00%)	9 (64.29%)/5 (35.71%)	3 (30.00%)/7 (70.00%)
Height (cm)^†^	161.6 (158.1, 165.17)	161.6 (156.4, 166.7)	161.7 (155.9, 167.4)
Weight (kg)^†^	62.46 (57.14, 67.78)	63.13 (56.26, 70.00)	61.52 (51.42, 71.62)
BMI (kg/m^2^)^†^	23.78 (22.20, 25.35)	24.04 (22.13, 25.95)	23.40 (20.26, 26.54)
Drinking (yes/no)^‡^	9 (37.50%)/15 (62.50%)	6 (42.86%)/8 (57.14%)	3 (30.00%)/7 (70.00%)
Smoking (yes/no)^‡^	3 (12.50%)/21 (87.50%)	2 (14.29%)/12 (75.71%)	1 (10.00%)/9 (90.00%)
Job (yes/no)^‡^	15 (62.50%)/9 (37.50%)	10 (71.43%)/4 (28.57%)	5 (50.00%)/5 (50.00%)
Duration of diseases (month)^†^	106.8 (57.0, 156.6)	95.1 (52.0, 138.2)	123.2 (5.7, 240.8)
The severity of asthma^‡^			
Intermittent mild		1 (7.1%)	
Persistent mild		7 (50.0%)	
Persistent moderate severity		4 (28.6%)	
Persistently severe		2 (14.3%)	
The severity of COPD^‡^			
(A)			2 (20.0%)
(B)			4 (40.0%)
(C)			4 (40.0%)
Use of bronchodilators or inhaled corticosteroids^‡^			
SABA	7 (29.17%)	7 (50.00%)	0 (0.00%)
LAMA	1 (4.17%)	0 (0.00%)	1 (10.00%)
LABA + ICS	7 (29.17%)	4 (28.57%)	3 (30.00%)
LABA + LAMA + ICS	1 (4.17%)	0 (0.00%)	1 (10.00%)
Pattern identification for asthma^‡^			
Wind-cold		3 (21.43%)	
Phlegm-dampness stagnation		1 (7.14%)	
Heart-kidney deficiency		9 (64.29%)	
Upper excess and lower deficiency		1 (7.14%)	
Pattern identification for COPD^‡^			
Wind-cold			2 (20.00%)
Kidney-yang deficiency			7 (70.00%)
Kidney-yin deficiency			1 (10.00%)

^†^Student's independent *t*-test, ^‡^Fisher's exact test, SABA: short-acting beta agonists; LABA: long-acting beta agonists; LAMA: long-acting muscarinic antagonists; ICS: inhaled corticosteroids.

**Table 2:** The number of participants treated with concomitant Korean medicine treatments.

	Acupuncture	Pharmacoacupuncture	Moxibustion
Baseline	20	10	8
Week 4	17	6	6
Week 8	8	5	4
Week 12	12	3	2

**Table 3:** Pulmonary function tests and PEFR before and after herbal medicine treatments.

	Total (*n* = 24)	Asthma (*n* = 14)	COPD (*n* = 10)
FVC			
Baseline	86.42 (78.08, 94.76)	88.00 (78.18, 97.82)	84.20 (67.13, 101.27)
Week 12	86.72 (76.543, 96.89)	88.00 (76.75, 99.24)	84.93 (65.62, 104.23)
*p* value	0.9326	0.9994	0.8227
FEV1			
Baseline	73.92 (64.26, 83.57)	79.93 (67.72, 92.14)	65.50 (48.63, 82.37)
Week 12	71.61 (60.67, 82.55)	77.68 (64.08, 91.27)	63.11 (47.41, 78.80)
*p* value	0.5987	0.7383	0.4743
FEV1/FVC			
Baseline	62.13 (56.32, 67.93)	67.29 (61.37, 73.21)	54.90 (44.00, 65.80)
Week 12	60.30 (53.83, 66.77)	65.50 (57.98, 73.03)	53.02 (42.92, 63.12)
*p* value	0.3256	0.5021	0.4523
PEFR			
Baseline	255.0 (209.7, 300.3)	286.4 (224.4, 348.5)	211.0 (143.1, 278.9)
Week 4	254.0 (212.5, 295.5)	280.4 (219.3, 341.4)	217.0 (165.2, 268.8)
*p* value	0.9424	0.7959	0.6327
Week 8	263.0 (226.3, 299.8)	293.8 (243.7, 343.9)	220.0 (170.4, 269.6)
*p* value	0.5750	0.7362	0.6099
Week 12	256.2 (218.6, 293.9)	276.4 (220.4, 332.3)	228.1 (187.5, 268.7)
*p* value	0.9305	0.6451	0.2201

Data are presented as mean (95% CI). *p* value by paired *t*-test.

**Table 4:** mCAMSOM-V before and after herbal medicine treatments.

	Total (*n* = 24)	Asthma (*n* = 14)	COPD (*n* = 10)
Baseline	19.04 (14.72, 23.37)	22.86 (17.45, 28.27)	13.70 (7.03, 20.37)
Week 12	17.33 (13.77, 20.90)	20.06 (15.55, 24.57)	13.52 (8.32, 18.71)
*p* value	0.0687	0.0401^*∗*^	0.8783

Data are presented as mean (95% CI). *p* value by paired *t*-test (^*∗*^*p* < 0.05).

**Table 5:** ACT or CAT before and after herbal medicine treatments.

	Asthma (*n* = 14)	COPD (*n* = 10)
Baseline	14.57 (11.92, 17.22)	17.70 (12.01, 23.39)
Week 4	17.39 (14.43, 20.34)	16.80 (11.21, 22.39)
*p* value	0.0603	0.6015
Week 8	17.33 (14.42, 20.23)	17.50 (12.91, 22.09)
*p* value	0.1053	0.9183
Week 12	16.26 (12.75, 19.76)	17.09 (9.89, 24.28)
*p* value	0.3032	0.7882

Data are presented as mean (95% CI). *p* value by paired *t*-test.

**Table 6:** Modified Borg Scale and mMRC before and after herbal medicine treatments.

	Total (*n* = 24)	Asthma (*n* = 14)	COPD (*n* = 10)
Modified Borg scale			
Baseline	2.17 (1.29, 3.04)	2.14 (0.72, 3.57)	2.20 (1.16, 3.24)
Week 4	1.50 (0.99, 2.02)	1.29 (0.53, 2.05)	1.80 (1.06, 2.54)
*p* value	0.0829	0.1747	0.2100
Week 8	1.86 (1.20, 2.52)	1.62 (0.84, 2.39)	2.20 (-0.86, 3.54)
*p* value	0.4233	0.3959	0.9999
Week 12	2.11 (1.47, 2.74)	2.26 (1.23, 3.29)	1.89 (1.14, 2.65)
*p* value	0.8585	0.8368	0.3195
mMRC			
Baseline	1.29 (0.91, 1.68)	1.07 (0.72, 1.43)	1.60 (0.76, 2.44)
Week 4	1.09 (0.80, 1.39)	0.81 (0.48, 1.13)	1.50 (0.99, 2.01)
*p* value	0.2566	0.0562	0.7976
Week 8	1.18 (0.85, 1.52)	0.95 (0.60, 1.31)	1.50 (0.80, 2.20)
*p* value	0.5389	0.5426	0.7804
Week 12	0.78 (0.51, 1.06)	0.71 (0.35, 1.07)	0.89 (0.49, 1.30)
*p* value	0.0067^*∗∗*^	0.0910	0.0269^*∗*^

Data are presented as mean (95% CI). *p* value by paired *t*-test (^*∗*^*p* < 0.05, ^*∗∗*^*p* < 0.01).

**Table 7:** SGRQ before and after herbal medicine treatments.

	Total (*n* = 24)	Asthma (*n* = 14)	COPD (*n* = 10)
Symptom			
Baseline	62.91 (55.81, 70.00)	70.73 (63.52, 77.93)	51.95 (40.25, 63.66)
Week 12	56.26 (47.36, 65.17)	63.38 (51.24, 75.53)	46.30 (35.50, 57.09)
*p* value	0.0732	0.1527	0.3084
Activity			
Baseline	47.41 (38.54, 56.28)	44.14 (34.40, 53.89)	51.99 (33.37, 70.60)
Week 12	45.35 (33.78, 56.92)	47.06 (30.98, 63.15)	42.95 (26.46, 59.44)
*p* value	0.6796	0.6514	0.2153
Impact			
Baseline	30.66 (23.20, 38.13)	34.94 (25.12, 44.77)	24.67 (12.04, 37.30)
Week 12	27.00 (19.02, 34.98)	34.50 (23.71, 45.28)	16.51 (6.88, 26.14)
*p* value	0.1066	0.8903	0.0147^*∗*^
Total			
Baseline	46.99 (40.77, 53.22)	49.94 (41.97, 57.91)	42.87 (31.62, 54.12)
Week 12	42.87 (34.76, 50.98)	48.32 (37.01, 59.62)	35.25 (25.23, 45.26)
*p* value	0.1190	0.6574	0.0265^*∗*^

Data are presented as mean (95% CI). *p* value by paired *t*-test (^*∗*^*p* < 0.05).

**Table 8:** Biomarkers before and after herbal medicine treatments.

	Total (*n* = 22)	Asthma (*n* = 12)	COPD (*n* = 10)
TNF-*α*			
Baseline	8.89 (−3.14, 20.92)	12.32 (−8.14, 32.79)	3.55 (−0.90, 8.00)
Week 12	2.56 (1.50, 3.62)	1.79 (0.89, 2.69)	3.75 (1.67, 5.83)
*p* value	0.2751	0.2649	0.8899
IL-4			
Baseline	4.59 (4.11, 5.07)	4.66 (4.06, 5.25)	4.48 (3.48, 5.49)
Week 12	5.09 (4.30, 5.88)	4.74 (3.88, 5.59)	5.64 (4.26, 7.03)
*p* value	0.2323	0.8526	0.1290
IL-5			
Baseline	0.99 (0.62, 1.36)	1.06 (0.46, 1.67)	0.88 (0.48, 1.27)
Week 12	1.53 (0.84, 2.22)	1.23 (0.30, 2.16)	2.00 (0.95, 3.04)
*p* value	0.2075	0.7700	0.0675
IL-6			
Baseline	1.26 (-0.67, 3.20)	1.63 (-1.68, 4.94)	0.69 (-0.13, 1.51)
Week 12	0.38 (0.18, 0.59)	0.31 (0.07, 0.55)	0.50 (0.11, 0.89)
*p* value	0.3524	0.3957	0.4763
IL-13			
Baseline	27.77 (17.45, 38.10)	27.96 (17.88, 38.03)	27.48 (2.30, 52.66)
Week 12	15.13 (6.03, 24.24)	15.61 (1.16, 30.05)	14.40 (5.30, 23.50)
*p* value	0.0411^*∗*^	0.1182	0.2267
CRP			
Baseline	0.203 (0.069, 0.337)	0.098 (0.036, 0.160)	0.350 (0.031, 0.669)
Week 12	0.387 (0.070, 0.705)	0.218 (0.035, 0.442)	0.625 (0.000, 1.320)
*p* value	0.1768	0.3129	0.3455
Fibrinogen			
Baseline	394.3 (328.8, 459.7)	353.6 (287.2, 419.9)	451.2 (315.2, 587.2)
Week 12	367.2 (324.3, 410.1)	336.4 (295.5, 377.3)	410.3 (332.5, 488.1)
*p* value	0.2901	0.5115	0.4103
IgE			
Baseline	173.0 (74.6, 271.3)	220.7 (56.0, 385.3)	106.2 (25.5, 186.8)
Week 12	158.6 (77.1, 240.0)	195.1 (64.0, 326.2)	107.4 (44.8, 170.3)
*p* value	0.1281	0.0541	0.9186

Data are presented as mean (95% CI). *p* value by paired *t*-test (^*∗*^*p* < 0.05).

## Data Availability

The datasets used or analyzed during the current study will be available from the corresponding authors on reasonable request.

## Abbreviations

ACT: Asthma control test
CAM: Complementary or alternative medicine
CAT: COPD assessment test
COPD: Chronic obstructive pulmonary disease
CRP: C-reactive protein
FEV1: Forced expiratory volume in 1 s
FVC: Forced volume capacity
GINA: Global Initiative for Asthma
GOLD: Global Initiative for Chronic Obstructive Lung Disease
IgE: Immunoglobulin E
IL: Interleukin
IMPSS: Integrative Medicine Patient Satisfaction Scale
mCAMSOM-V: Modified Clinical Asthma Measurement Scale in Oriental Medicine-V
mMRC: Modified Medical Research Council
PEFR: Peak expiratory flow rate
PFT: Pulmonary function test
SGRQ: St. George's Respiratory Questionnaire
TNF-*α*: Tumor necrosis factor-*α*.
